# Fat Taste Sensitivity Is Associated with Short-Term and Habitual Fat Intake

**DOI:** 10.3390/nu9070781

**Published:** 2017-07-20

**Authors:** Andrew Costanzo, Liliana Orellana, Caryl Nowson, Konsta Duesing, Russell Keast

**Affiliations:** 1 Centre for Advanced Sensory Sciences, School of Exercise and Nutrition Sciences, Deakin University, 1 Gheringhap Street, Geelong 3220, Australia; andrew.costanzo@deakin.edu.au; 2 Biostatistics Unit, Faculty of Health, Deakin University, 1 Gheringhap Street, Geelong 3220, Australia; l.orellana@deakin.edu.au; 3 Institute of Physical Activity and Nutrition, School of Exercise and Nutrition Sciences, Deakin University, 1 Gheringhap Street, Geelong 3220, Australia; caryl.nowson@deakin.edu.au; 4 CSIRO Health & Biosecurity, North Ryde 2113, Australia; konsta.duesing@csiro.au

**Keywords:** fat taste, sensitivity, taste thresholds, fat intake, BMI, liking, women

## Abstract

Evidence suggests individuals less sensitive to fat taste (high fat taste thresholds (FTT)) may be overweight or obese and consume greater amounts of dietary fat than more sensitive individuals. The aims of this study were to assess associations between FTT, anthropometric measurements, fat intake, and liking of fatty foods. FTT was assessed in 69 Australian females (mean age 41.3 (15.6) (SD) years and mean body mass index 26.3 (5.7) kg/m^2^) by a 3-alternate forced choice methodology and transformed to an ordinal scale (FT rank). Food liking was assessed by hedonic ratings of high-fat and reduced-fat foods, and a 24-h food recall and food frequency questionnaire was completed. Linear mixed regression models were fitted. FT rank was associated with dietary % energy from fat ($\hat{\beta }$ = 0.110 [95% CI: 0.003, 0.216]), % energy from carbohydrate ($\hat{\beta }$ = −0.112 [−0.188, −0.035]), and frequency of consumption of foods per day from food groups: high-fat dairy ($\hat{\beta }$ = 1.091 [0.106, 2.242]), meat & meat alternatives ($\hat{\beta }$ = 0.669 [0.168, 1.170]), and grain & cereals ($\hat{\beta }$ = 0.771 [0.212, 1.329]) (adjusted for energy and age). There were no associations between FT rank and anthropometric measurements or hedonic ratings. Therefore, fat taste sensitivity appears to be associated with short-term fat intake, but not body size in this group of females.

## 1. Introduction

Dietary fat is an energy-dense macronutrient that contributes to approximately 31–32% of energy intake in Australian adult diets [1]. Passive overconsumption of dietary fats is common due to their palatability, which is a major contributor to the development of overweight and obesity. Oral perception of fatty acids (fat taste) has recently been recognised as a primary taste, which may have a regulatory role in the consumption of dietary fat in humans [2,3,4]. For fat taste to be initiated, free fatty acids (FFAs) must activate fat taste receptors located on taste cells [5]. FFAs occur in small amounts in fatty foods [6], and human lingual lipases may also increase FFA exposure in the oral cavity by hydrolysing triacylglycerides (TAGs) [7]. In large amounts, FFAs elicit a rancid taste to prevent the consumption of spoiled lipids in foods. It should be noted that FFA taste differs from triacylglyceride (TAG) perception, which imparts odour and textural dimensions presumably independent of the fatty acid taste dimension.

Cross-sectional studies have shown that fat taste sensitivity is associated with the consumption of fatty foods, such that those with a lower sensitivity tend to consume larger amounts of dietary fat. Furthermore, there is some evidence that those with a lower sensitivity to fat taste are more likely to have a higher body mass index (BMI) [8,9,10,11,12]. However, the link between fat taste and BMI is contentious, as many studies have been unable to find an association [13,14,15,16]. A recent meta-analysis of seven cross-sectional studies found that sensitivity to fat taste does not contribute to or result from obesity [17].

Fat taste thresholds are the current standard for measuring sensitivity to fat taste, in that sensitivity decreases as fat taste thresholds increase [18]. Fat taste thresholds are defined as the lowest amount of FFA necessary to produce a detectable taste response. There are large variations in fat taste thresholds among the population [14,15] possibly due to variations in habitual fat intake. Two intervention studies aiming to assess the link between fat intake and fat taste thresholds both showed that fat taste thresholds decreased when participants were exposed to a low-fat diet over a four-week [15] and six-week [19] period. In addition, fat taste thresholds increased in healthy weight participants exposed to a high-fat diet over a four-week period [15], but there was no increase in fat taste threshold in overweight or obese individuals, presumably because their fat taste sensitivity was already impaired. However it remains uncertain whether the change in thresholds are specific to total fat intake or related to weight fluctuations as participants in both studies did not maintain their baseline weight, losing weight on the low-fat diet and gaining weight on the high-fat diet [15,19]. 

The ability to discriminate foods based on perception of TAGs also has health implications as it can allow individuals to make food choices which are lower in dietary fats. TAG perception varies widely amongst the population [11,20] and limited evidence suggests an association between fat taste sensitivity and TAG perception [10,11,15]. Since fat taste is only the perception of FFAs in the oral cavity, this association is likely to be unrelated to the TAGs themselves, but may instead be due to naturally occurring FFAs in fatty foods [6] or hydrolysis of TAGs into FFAs by lingual lipase [7]. Similarly, obese individuals [21] or individuals with a larger waist circumference [20] have been found to have impaired perception of TAGs compared to healthy weight individuals. 

Liking fatty foods is also a promoter of dietary fat consumption [9], although the current evidence for the relationship between fat perception and health outcomes is weak [22]. There may be a relationship between liking of fatty foods and fat taste sensitivity, with sensitive individuals preferring low-fat foods compared to less sensitive individuals [9,13]. This is likely due to the unpleasant rancid taste of FFAs found in high-fat foods [6]. 

The aims of this study were to assess the associations between fat taste thresholds, anthropometric measurements, fat intake, and liking of fatty foods in a sample of Australian adult women. It was hypothesised that fat taste thresholds would be positively associated with fat intake and liking of high-fat foods and not associated with level of obesity.

## 2. Materials and Methods

### 2.1. Participants

Sixty-six twin pairs (132 individuals) were screened by Twins Research Australia to participate in a randomised controlled trial (RCT) assessing the impact of alterations in dietary fat intake and heritability on fat taste function. Monozygotic (MZ) and dizygotic (DZ) pairs were included in this study. Participants were eligible if 18 to 68 years old, did not have dairy allergies or intolerances or illnesses preventing them from eating foods included in the study, and they were neither pregnant nor lactating. Forty-six twin pairs (34 female pairs, 11 male pairs, and one gender discordant pair—92 individuals) were eligible for the RCT and completed baseline measurements.

The current study reports the results of the 69 females (34 female twin pairs—26 MZ pairs, 8 DZ pairs—and one individual from the gender-discordant pair) who completed baseline measurements of the RCT. In the 23 males (11 male twin pairs—10 MZ pairs, 1 DZ pair—and one individual from the gender-discordant pair), the main outcome, fat taste (FT) rank was heavily skewed in males with 10 of 23 men (43%) classified in the two more extreme categories (Figure S1). Male data clearly violated the assumption of the analytical approach selected (linear mixed model). For this reason, we report results only in women (*n* = 69). Male characteristics (Table S1) and associations with fat taste sensitivity (Table S2) have been included as supplementary data.

The study was conducted in accordance with the Declaration of Helsinki, the protocol was approved by the Deakin University Human Research Ethics Committee (2013-110), and written informed consent was obtained by all participants prior to participation. 

### 2.2. Study Outline

Each participants attended a 2-h laboratory session at the Centre for Advanced Sensory Science at Deakin University, Burwood, VIC. Tests were conducted in a temperature and sound controlled partitioned sensory booths using computer software Compusense Cloud as part of the Compusense Academic Consortium (Compusense Inc., Guelph, ON, Canada). A 15 min break was provided in the middle of each session to prevent fatigue. Participants were asked to avoid eating or drinking anything but water and to avoid brushing their teeth or using mouthwash up to an hour prior to their tasting session. Tasting sessions measured for (1) detection thresholds to oleic acid (fat taste thresholds); (2) TAG ranking ability; (3) liking ratings for high-fat and reduced-fat foods; and (4) sensitivities to the five basic tastes [23]. Anthropometric measurements were at the beginning of the taste session. A 24-h food recall was collected during the session by a nutritionist. Approximately one week prior to the tasting session, participants were asked to complete a Food Frequency Questionnaire on their eating habits, which was completed at home.

### 2.3. Fat Taste Detection Thresholds

Detection thresholds to oleic acid (C18:1) were measured using established methods [23]. Food grade C18:1 was obtained from Sigma Aldrich (St. Louis, MO, USA) and was stored under nitrogen gas below 4 °C. C18:1 was added at varying concentrations (0.02, 0.06, 1.00, 1.40, 2.00, 2.80, 3.80, 5.00, 6.40, 8.00, 9.80, 12.00, and 20.00 mM) to long-life fat-free milk (Devondale, Southbank, VIC, Australia). All preparations were mixed with 5% (*w*/*v*) gum arabic (pre-hydrated FT Powder, TIC Gums, Alchemy Agencies, Parramatta, NSW, Australia) and 5% (*v*/*v*) liquid paraffin (Faulding Remedies, Virginia, QLD, Australia) to produce perceptually identical textural attributes including viscosity and lubricity between C18:1 and control samples. To prevent oxidation of C18:1, all samples were mixed with 0.01% *w*/*v* EDTA (Merck, Darmstadt, Germany). Samples were homogenised for 30 s/100 mL at 12,000 rpm (Silverston L4RT homogeniser, Longmeadow, MA, USA), prepared no more than 2 h prior to testing, and served at room temperature. Control samples were prepared in the same way, but without added C18:1. Participants were asked to rinse their mouths with water before beginning the task and between sample sets. To prevent confounding non-taste sensory inputs, participants wore nose clips and all tests were conducted under red light.

Detection thresholds for the fatty acids were determined using ascending series 3-alternate forced choice (3-AFC) methodology [23]. Participants were provided with multiple sample sets each containing three randomly-ordered samples per set, two controls and one containing C18:1. Participants were asked to taste each sample in the set and identify one as “different.” Correct identification of the C18:1 sample resulted in the participants repeating the same sample set. Incorrect identification of the C18:1 sample resulted in new sample set with a higher concentration of C18:1. This continues in an ascending order from the lowest (0.02 mM) to the highest (20 mM) concentration. End-point was defined as the concentration of C18:1 required for a participant to correctly identify the C18:1 in three consecutive sample sets of the same concentration, in line with commonly established sensory testing procedures [23]. 

In order to reduce sensory fatigue and to help recognition of fat taste, participants were provided with warm-up sets prior to the 3-AFC test. A warm-up set contained a C18:1 sample (initially 3.8 mM) and a control sample. If participants were unable to perceive a difference between the control and C18:1 sample during the first warm-up set, then they were provided with a new warm-up set at 8 mM. The 3-AFC test began with the highest C18:1 concentration sample set that could not be differentiated to the control during warm-up (the 3-AFC test began with 0.02 mM if participant was able to differentiate the 3.8 mM sample from the control sample). The 3-AFC test was performed in duplicate with participants eating a low-fat plain water cracker (Manassen Foods, Horsley Park, NSW, Australia) between tests to reduce sensory fatigue. Detection thresholds were defined as the mean of the two end-points, as two within-day measures has been shown to be reliable [24]. If the two end-points were greater than three concentration steps apart, the test was repeated for a third time, and the detection threshold was defined as the mean of the three end-points. If participants were unable to correctly identify the C18:1 sample at the highest concentration (20 mM) for at least one of their trials, then they were given a detection threshold of >20 mM. Of note, due to the range of concentrations of fatty acid tested (0.02, 0.06, 1.00, 1.40, 2.00, 2.80, 3.80, 5.00, 6.40, 8.00, 9.80, 12.00, and 20.00 mM), the outcome fat taste threshold is an interval censored variable; i.e., a threshold of 20 mM indicates that the participant’s actual threshold is anywhere between 12 and 20 mM. For this reason, fat taste threshold was transformed to an ordinal variable—FT rank—ranging from 0 to 12, with higher ranks implying lower sensitivity to fat taste (Table 1).

### 2.4. Fat Ranking Task

This task was designed to evaluate the participants’ ability to discriminate different levels of fat content between food samples [23]. Canola oil (Woolworths, Bella Vista, NSW, Australia) was added to low-fat custard (Parmalat, Brisbane, QLD, Australia) up to 2%, 6% and, 10% (*w*/*w*) oil in custard. A custard sample remained oil free (0%). All samples were stirred vigorously. All four custard samples were presented to participants in a randomised order, and participants were asked to taste each sample and rank them according to their fat content. The custard samples were served at room temperature and prepared no more than 2 h prior to testing. To prevent visual cues, the test was conducted under red light. Participant’s ranking ability scores was determined based on calculations proposed by Bolhuis et al. [25]. Accurately ranking samples in ascending or descending order are both considered to be correct; therefore, negative scores were converted to positive values. Final scores ranged from 0 to 10, with 10 being able to correctly rank the samples based on fat content, and 0 being unable.

### 2.5. 24-H Dietary Recall

A 24-h dietary recall was used to assess short-term dietary intake. A single three-pass 24-h dietary recall [26] of the day prior to the testing session was conducted by a trained nutritionist. Food recalls were analysed for energy intake (MJ), total consumption (g) of protein, fats (total fat, saturated fat, monounsaturated fat, and polyunsaturated fat), carbohydrates, and alcohol, and percentage of energy derived from protein, fats, and carbohydrates using computer software FoodWorks (version 8, Xyris, Spring Hill, QLD, Australia).

### 2.6. Food Liking

Liking of foods based on fat content was measured rating seven high-fat (HF) foods and seven reduced-fat (LF) counterparts. Participant liking was measured by rating “liking” on a hedonic general labelled magnitude scale with anchors −100 (extremely dislike), 100 (extremely like), and 0 (neither like nor dislike). Foods were presented under red light to reduce visual differences between samples. Foods measured included savoury biscuits (Arnott’s, Sydney, NSW, Australia), peanut butter (Mondelez, Melbourne, VIC, Australia), hummus (Black Swan, Clayton, VIC, Australia), salad dressing (Goodman Fielder, North Sydney, NSW, Australia), processed cheese (Mondelez, Melbourne, VIC, Australia), cream cheese (Mondelez, Melbourne, VIC, Australia), and chocolate mousse (Fonterra, Notting Hill, VIC, Australia). Foods were always presented in the order listed above to simulate normal eating behaviour. 

HF and LF liking scores were calculated as the mean of the seven HF foods and seven LF foods liking ratings, respectively. The differences between the HF and LF scores (HF-LF liking score) was also calculated to control for individual preferences for each food item. 

### 2.7. Five Basic Tastes

Participants rated the intensities of sweet, salty, sour, bitter, and umami solutions at concentrations prepared based on Webb et al. [27]. Concentrations were prepared at supra-threshold concentrations (weak, moderate, and strong), made using sucrose (Woolworths, Bella Vista, NSW, Australia), sodium chloride (Woolworths, Bella Vista, NSW, Australia), citric acid (Ward McKenzie, Altona, VIC, Australia), caffeine (Sigma-Aldrich, St. Louis, MO, USA) and monosodium glutamate (MSG) (Ajinomoto Cooperation, Tokyo, Japan), respectively. To prevent confounding non-taste sensory inputs, participants wore nose clips and the test was conducted under red light. Participants tasted each sample and rated the intensities on a general labelled magnitude scale with anchors 0 (no taste) to 100 (strongest imaginable taste). All solutions were prepared within five days of testing, stored at 4 °C, and served at room temperature. Participants were presented all three concentrations (weak, moderate and strong) of one taste at a time in random order. The sequence of the five basic taste tests was also randomised. 

### 2.8. Anthropometry

Body weight was measured after removal of shoes, heavy clothing, and any items in their pockets using electronic scales (OHAUS NV4101, Parsippany, NJ, USA), and height was measured using a free-standing stadiometer (SECA, Hamburg, Germany). BMI was calculated as weight (kg)/height (m)^2^. Hip and natural waist (midway between the lowest rib and the iliac crest) circumferences were measured according to World Health Organisation guidelines [28]. 

### 2.9. Food Frequency Questionnaire

A Food Frequency Questionnaire (FFQ) adapted from the 1995 Australian National Nutrition Survey FFQ [29] was used to assess habitual pattern of food intake. Participants were required to indicate on average, how many times in the previous month they consumed 96 different food or beverage items. In total, nine categories of food were assessed including dairy products; breads and cereals; meat, fish and eggs; other offal; sweets, baked goods and snacks; dressings; non-dairy beverages; vegetables; and fruits. The frequency they could be consumed ranged from “never or less than once a month” to “six or more times per day”. Each frequency category was converted into a daily equivalent value for occasions of consumption: for example, “never, or less than once a month” = 0.02, “one to three times per month” = 0.07, “once per week” = 0.1, “two to four times per week” = 0.4, “five to six times per week” = 0.8, “once per day” = 1.0, “two to three times per day” = 2.5, “four to five times per day” = 4.5, and “six plus times per day” = 6 [30]. 

For analysis, food items were categorised into food groups based on the classification system used in the 2011–2013 Australian Health Survey [30], which used a hierarchical numeric system to classify foods. In this system, individual food and beverage items are assigned an eight-digit food ID where two- and three-digit food groups describe major and sub-major food groups, respectively. All food items from the FFQ were categorised based on two-digit food codes, except for dairy foods, which were categorised based on three-digit food codes to differentiate high-fat and low-fat sources of dairy.

The following major food groups were combined into a “meat & meat alternatives” category to reduce multiple comparisons, as they all contain significant amounts of dietary protein: “meat, poultry and game products and dishes”, “fish and seafood products and dishes”, “egg products and dishes”, “seed and nut products and dishes”, and “dairy and meat substitutes”. Dairy products were split into two categories: low-fat dairy and high-fat dairy based on their “sub-major food groups”. Any foods flagged as “discretionary” by the Australian Health Survey—Discretionary Food List were categorised into a “discretionary foods” category and not included in any other food group [31]. The final categories analysed included meat & meat alternatives, fruits, vegetables, high-fat dairy, low-fat dairy, grains & cereals, discretionary foods, and alcoholic beverages. A full list of the categorisation of each food item can be seen in Table S3. 

### 2.10. Statistical Analysis

Statistical analyses were conducted using statistics package SAS (v9.3, 3 SAS Institute, Cary, NC, USA). Null hypotheses were rejected at *p* < 0.05. Descriptive statistics are reported as mean and SD, and categorical data presented as *n* and %. Estimated coefficients obtained under linear mixed-effects models are reported along with 95% confidence intervals. Estimated coefficients obtained under linear mixed-effects models ($\hat{\beta }$) are reported along with 95% confidence intervals. $\hat{\beta }$ indicates the change in FT rank per unit of change in the independent variable after controlling for other variables in the model.

The association between FT rank and other variables was assessed using linear mixed-effects models including the variable of interest and age as fixed effects, and the twin pair as a random effect to account for the clustering induced by the twin. We included age as a covariate to account for any effect of age on taste. When considering associations between FT rank and nutrient intakes, we report the estimates from the model described above (unadjusted) and from a model with energy adjusted FT rank to control for differences in energy intake [32]. We also report associations between FT rank and nutrient energy as a percentage of total energy intake. Food frequency questionnaire food group analyses were also conducted with and without adjusting for energy intake. The associations between FT rank and nutrient intakes, as above, were also compared between underweight/healthy weight individuals (*n* = 36) and overweight/obese individuals (*n* = 33) using the same linear mixed model including BMI status as a fixed effect.

## 3. Results

Characteristics of the females (*n* = 69) who were assessed in this study are detailed in Table 2. For fat taste thresholds, all inter-individual 3-AFC test end-points were within three concentration steps apart for the first two measurements. Therefore, no individuals in this study needed to complete a third 3-AFC test.

### 3.1. 24-H Dietary Recall

Sixteen participants were classified as low-energy reporters according to Goldberg cut-off values [34]. The following analyses were conducted with and without low-energy reporters. Estimated coefficients and conclusions were similar regardless of low-energy reporter exclusion (Table S4). Therefore, we report the analyses using the entire female sample.

Energy and nutrient intakes of the females who were assessed in this study are detailed in Table 3.

There was no significant association between FT rank and energy intake (MJ) (Table 4). After adjusting for energy intake, there was a significant positive association between FT rank and percent (%) energy from fat, and a significant negative association between % energy from carbohydrate (Table 4). For example, a 10% increase in energy from total fat would be associated with a 1.10 unit change in FT rank. In addition, there were positive associations that approached significance between FT rank, % energy from monounsaturated fat (*p* = 0.067) and % energy from saturated fat (*p* = 0.100). When assessing participants split by BMI status group (underweight/healthy weight versus overweight/obese), there was no significant effect of BMI status on the associations between FT rank and any of the macronutrient intakes. 

### 3.2. Food Frequency Questionnaire

The FFQ assessed self-reported habitual food consumption of specific food items. After adjusting for energy intake, there were significant positive associations between FT rank and daily consumption of meat & meat alternatives, HF dairy and grain & cereal (Table 5). 

### 3.3. Food Liking

Liking scores of the seven LF and HF foods (savoury biscuits, peanut butter, hummus, salad dressing, processed cheese, cream cheese, and chocolate mousse) were rated from −100 to 100. LF and HF liking scores were calculated as the mean of the seven LF and HF food liking ratings, respectively. The mean LF liking score was 13.6 (11.3), HF liking score was 20.6 (11.6) and the difference (HF-LF) liking score was 7.0 (7.6). There were no associations between FT rank and LF liking score ($\hat{\beta }$ = 0.025 [95% CI: −0.039, 0.088]), HF liking score ($\hat{\beta }$ = −0.004 [−0.061, 0.053]) or HF-LF liking score ($\hat{\beta }$ = −0.009 [−0.023, 0.005]). 

### 3.4. Fat Ranking Task

The possible scores for the fat ranking task ranged from 0–10, with 10 being able to fully discriminate the fat content between the samples from lowest to highest. The mean score for the fat ranking task was 5.8 (3.7) (SD). No association was observed between fat ranking task score and FT rank ($\hat{\beta }$ = −0.049 [−0.282, 0.184]). 

### 3.5. Five Basic Tastes

No significant associations were observed between FT rank and sensitivity to any of the five basic tastes (sweet: $\hat{\beta }$ = 0.184 [−2.224, 2.968]; salty: $\hat{\beta }$ = 0.143 [−2.683, 2.968]; sour: $\hat{\beta }$ = −1.449 [−4.703, 1.805]; bitter: $\hat{\beta }$ = −2.198 [−5.054, 0.657]; umami: $\hat{\beta }$ = −0.511 [−3.144, 2.123]).

### 3.6. Anthropometry

There were no associations between FT rank and BMI ($\hat{\beta }$ = 0.077 [−0.171, 0.325]), waist circumference ($\hat{\beta }$ = 0.040 [−0.034, 0.114]) or waist-hip ratio ($\hat{\beta }$ = 0.488 [−12.671, 13.647]).

## 4. Discussion

The current study assessed the associations between FT rank, anthropometric measurements, fat intake, and liking of fatty foods in healthy Australian adult women. There were no associations between FT rank, anthropometric measurements and liking of fatty foods. There were positive associations between FT rank and fat intake, only when expressed as % energy from fat, and a negative association between FT rank and % energy from carbohydrate. Habitual consumption of meat & meat-alternatives, high-fat dairy, and grain & cereal were positively associated with FT rank. There was no association between FT rank and total dietary fat intake (g), with or without controlling for energy. This indicates that fat taste sensitivity is associated with the proportion of fat consumed relative to total energy intake rather than the total amount of fat consumed. It should be noted that the nutrient intakes in this sample of females was reflective of Australian female diets, as energy from macronutrients were similar to what was found in the Australian Health Survey 2011–2012 [1].

The literature surrounding the relationship between fat taste sensitivity and adiposity is mixed. Many studies have reported a negative association between fat taste sensitivity and BMI [8,9,10,11,12], while others reported no association [13,14,15,16]. Methodologies used in these studies are similar, and reasons for the differing results are not clear. A recent meta-analysis of seven cross-sectional studies clearly demonstrated that fat taste sensitivity was not associated with BMI [17]. The results from the current study match the meta-analysis, in that BMI was not associated with fat taste sensitivity. Similarly, we found no association between waist circumference or waist-hip ratio with fat taste sensitivity. 

Despite impaired fat taste sensitivity having no association with BMI, it may still have implications for negative health outcomes. The current Australian and international dietary recommendations are to reduce saturated fat intake and increase polyunsaturated fat intake [35,36]. In the current study, associations between FT rank, % energy of saturated, and % energy of monounsaturated fat were in the expected direction (positive), although they did not reach statistical significance. Tucker et al. [37] previously reported a correlation between saturated fat and fat taste sensitivity, although not for monounsaturated fat. The associations between FT rank and polyunsaturated fat, after adjusting for both intake and percentage of energy, were negligible. If impaired fat taste sensitivity does contribute to increased saturated fat intake, then understanding the factors that influence fat taste sensitivity is important.

The food groups associated with fat taste sensitivity were also assessed in this study. Frequency of consumption of foods per day from high-fat dairy, grain & cereal, and meat & meat alternative food groups, was associated with higher FT rank. Increasing the consumption of high-fat dairy, grain & cereal, or meat & meat alternative foods in one occasion per day is associated with increases of 1.1, 0.8, and 0.7 units in FT rank, respectively. The large increase in FT rank associated with consumption of high-fat dairy matches the macronutrient intake data from this study, as high-fat dairy is a major source of saturated and monounsaturated fat. However, the association with meat & meat alternatives is harder to understand, as there was no association between FT rank and protein intake. It is likely that the association between FT rank and meat & meat alternatives is due to fat in meat products. While the Australian Guide to Healthy Eating [34] recommends consumption of lean sources of protein, the FFQ used was not able to differentiate whether meat items was lean or not (for example, “Mixed dishes with beef, veal—e.g., casserole, stir-fry”). Therefore, we could not split meat & meat alternatives into high-fat and low-fat categories as we did with dairy foods. However, previous research has shown that regularly trimming fat off meat prior to consumption is positively associated with fat taste sensitivity [11]. The association between FT rank and grain & cereal consumption is also difficult to understand because there was a negative association between FT rank and carbohydrate intake. In an effort to understand this, we assessed the different food items that fell into this category to see if any of the items were responsible for this association. Of the 9 food items, only “white bread, toast or rolls” had a significant association with FT rank ($\hat{\beta }$ = 1.623 [0.243, 3.003] *p* < 0.05). We assume that this association may be due to the addition of butter and other fatty spreads that are commonly consumed with white bread, and not due to the bread itself. This is supported by Stewart et al. [11], as it was found that consumption of high fat spreads was negatively associated with fat taste sensitivity. When “white bread, toast, or rolls” is omitted from the grain & cereal category, it is no longer significantly associated with FT rank. 

In the current study, it was found that liking fatty foods is not associated with fat taste sensitivity. This has been demonstrated in previous studies [15,19], although there is some evidence that less sensitive individuals have a higher preference for fatty foods [8,25]. This is interesting, as it suggests that hedonics are not drivers for fat intake with regard to fat taste sensitivity. Similarly, fat perception had no association with fat taste sensitivity. It is likely that the main driver of fat intake in less sensitive individuals is a reduced satiety response to fatty foods, both from impaired taste signalling and the reduced gastrointestinal tract [12].

There are limitations that should be acknowledged for this study. As this is a secondary analysis of baseline data of the main trial, sample size was calculated for the trial, and therefore the study is underpowered to detect small associations between BMI and fat taste threshold. However, our sample size is comparable to other similar studies that assessed the association between fat taste sensitivity and BMI [8,10,11,13,37]. Macronutrient intake assessment was based on one 24-h recall prior to the day of testing. A 24-h recall is subject to memory and participant biases, and it only provides an estimate of one day’s intake. The FFQ was not sufficiently detailed to enable the discrimination of lean or fatty types of meat. Also, phase of menstrual cycle was not controlled for, which may have an effect on fat taste sensitivity, as it has been shown that taste function increases at the time of menstruation in females [38].

Evidence from literature surrounding fat taste sensitivity and adiposity, food consumption, hedonics, and TAG perception is mixed. The current study is supportive of findings from recent studies regarding fat taste, in that there is no association between fat taste sensitivity and BMI, hedonics, and TAG perception, although short-term and habitual fat intake is associated with higher fat taste sensitivity, particularly saturated and monounsaturated fat.

## Figures and Tables

**Table 1:** Fat taste ranks and their corresponding fat taste threshold concentration ranges.

Fat Taste Threshold (mM)	Fat Taste Rank
0.02–0.99	0
1.00–1.39	1
1.40–1.99	2
2.00–2.79	3
2.80–3.79	4
3.80–4.99	5
5.00–6.39	6
6.40–7.99	7
8.00–9.79	8
9.80–11.99	9
12.00–19.99	10
20.00	11
>20.00	12

**Table 2:** Characteristics of study participants.

Characteristic	Mean	SD
Age (years) (range 18–62 years)	41.3	15.6
Height (cm)	163.1	7.8
Weight (kg)	70.1	16.7
BMI (kg/m^2^)	26.3	5.7
Waist Circumference (cm)	82.2	15.3
Waist-Hip Ratio	0.80	0.07
Fat Taste Rank	6.3	3.4
Weight Status	*n*	%
Underweight	3	4.3%
Healthy Weight	33	47.8%
Overweight	13	18.8%
Obese	20	29.0%

Underweight BMI ≤ 18.5 kg/m^2^; healthy weight BMI = 18.5–24.9 kg/m^2^; overweight BMI = 25–29.9 kg/m^2^; obese BMI ≥ 30 kg/m^2^ [33].

**Table 3:** Energy and nutrient intakes of study participants from the 24-h food recall.

	Nutrient Intake (g)	% Energy from Nutrients
*n* = 69	Mean (SD)	Mean (SD)
Energy (MJ)	7.8 (2.8)	-
Total Fat	73.5 (33.4)	34.2 (7.5)
Sat. Fat	28.1 (13.8)	13.0 (3.6)
Mono. Fat	28.2 (13.8)	13.1 (3.6)
Poly. Fat	11.0 (7.9)	5.2 (2.6)
Protein	90.7 (36.9)	20.2 (5.3)
CHO	189.4 (76.5)	40.3 (9.1)
Alcohol	4.7 (13.9)	1.7 (5.2)

Sat. Fat, saturated fat; Mono. Fat, monounsaturated fat; Poly. Fat, polyunsaturated fat; CHO, carbohydrate.

**Table 4:** Associations between fat taste (FT) rank, energy, and macronutrient intakes from the 24-h food recall.

	Nutrient Intake (g)	Nutrient Intake (g): Adjusted for Energy	% Energy from Nutrients
*n* = 69	$\hat{\beta }$ (95% CI)	$\hat{\beta }$ (95% CI)	$\hat{\beta }$ (95% CI)
Energy (MJ)	0.1 (−0.1, 0.3)	-	-
Total Fat	0.015 (−0.010, 0.041)	0.017 (−0.008, 0.041)	0.110 (0.003, 0.216) *
Sat. Fat	0.045 (−0.018, 0.107)	0.051 (−0.013, 0.115)	0.204 (−0.040, 0.448)
Mono. Fat	0.042 (−0.015, 0.099)	0.044 (−0.012, 0.101)	0.216 (−0.015, 0.446)
Poly. Fat	−0.005 (−0.146, 0.136)	−0.005 (−0.133, 0.123)	0.063 (−0.424, 0.549)
Protein	0.012 (−0.009, 0.032)	0.012 (−0.009, 0.032)	0.077 (−0.073, 0.227)
CHO	−0.002 (−0.011, 0.006)	−0.004 (−0.011, 0.004)	−0.112 (−0.188, −0.035) **
Alcohol	0.022 (−0.015, 0.060)	0.021 (−0.018, 0.060)	0.075 (−0.028, 0.178)

$\hat{\beta }$, estimated coefficient obtained under a mixed model including twin pair as a random effect; regression analysis was adjusted for age; CI, confidence interval; Sat. Fat, saturated fat; Mono. Fat, monounsaturated fat; Poly. Fat, polyunsaturated fat; CHO, carbohydrate; * *p*-value < 0.05; ** *p*-value < 0.01.

**Table 5:** Associations between fat taste thresholds and frequency of food group consumption as reported by the Food Frequency Questionnaire (FFQ).

	Self-Reported Consumption (Occasions Of Consumption/Day)	Self-Reported Consumption Adjusted for Energy Intake (Occasions of Consumption/Day)
*n* = 69	$\hat{\beta }$ (95% CI)	$\hat{\beta }$ (95% CI)
Meat & Meat Alternatives	0.616 (0.102, 1.130) *	0.669 (0.168, 1.170) **
Fruit	−0.485 (−1.024, 0.054)	−0.344 (−0.841, 0.154)
Vegetable	−0.039 (−0.226, 0.148)	−0.023 (−0.204, 0.157)
LF Dairy	−0.018 (−0.502, 0.466)	−0.011 (−0.476, 0.455)
HF Dairy	1.033 (−0.223, 2.288)	1.091 (0.106, 2.242) *
Grains & Cereal	0.717 (0.152, 1.282) *	0.771 (0.212, 1.329) **
Discretionary Food	−0.129 (−0.581, 0.324)	−0.158 (−0.615, 0.299)
Alcoholic Beverage	0.614 (−0.711, 1.940)	−0.067 (−1.487, 1.354)

$\hat{\beta }$, estimated coefficient obtained under a mixed model including twin pair as a random effect; regression analysis was adjusted for age; CI, confidence interval; HF, high fat; LF, low fat; * *p*-value < 0.05; ** *p*-value < 0.01.

## Supplementary Materials

The following are available online at www.mdpi.com/2072-6643/9/7/781/s1, Figure S1: Distribution of FT Rank for male and female participants, Table S1: Characteristics of excluded male participants, Table S2: Associations between FT rank, energy, and macronutrient intakes from the 24-h food recall in excluded male participants, Table S3: Categories for food items assessed in the Food Frequency Questionnaire based on the AHS 2011–2013 classification system, Table S4: Associations between FT rank, energy, and macronutrient intakes in women excluding low-energy reporters.
