# Supplementary material for: Fat Taste Sensitivity Is Associated with Short-Term and Habitual Fat Intake
Source: Nutrients. 2017 Jul 20;9(7):781. doi: 10.3390/nu9070781 (PMC5537895; doi:10.3390/nu9070781)
Supplement: Supplementary file 1 [file nutrients-09-00781-s001.docx]

**Figure S1.** Distribution of FT Rank for male and female participants


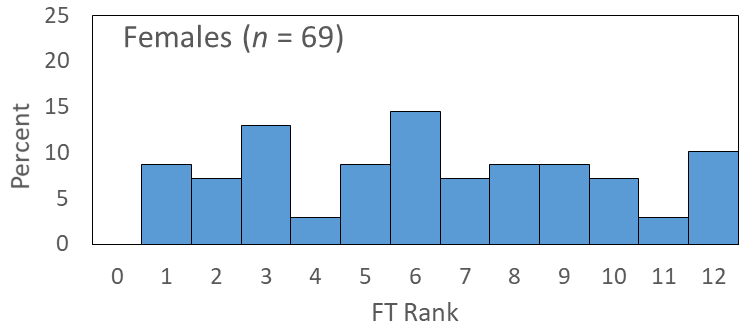

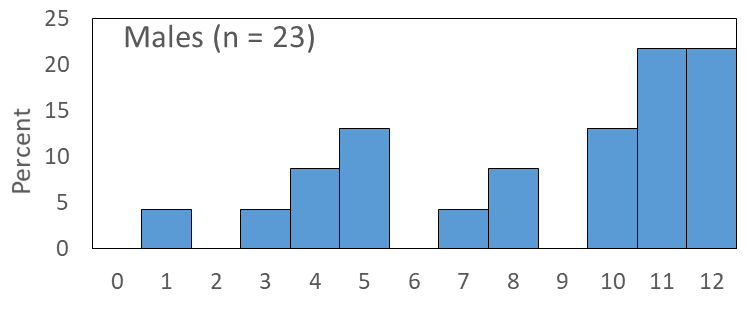


**Table S1**. Characteristics of excluded male (*n*=23) participants

| Characteristic | Mean | SD |
| --- | --- | --- |
| Age (years) (range 20-68 years) | 46.4 | 16.7 |
| Height (cm) | 175.7 | 7.8 |
| Weight (kg) | 85.2 | 15.7 |
| BMI (kg/m^2^) | 27.7 | 5.0 |
| Waist Circumference (cm) | 94.5 | 14.1 |
| Waist-Hip Ratio | 0.92 | 0.08 |
| Fat Taste Rank | 8.5 | 3.5 |
| Weight Status | *n* | % |
| Underweight | 0 | 0% |
| Healthy Weight | 7 | 30.4% |
| Overweight | 11 | 47.9% |
| Obese | 5 | 21.7% |

Underweight BMI = < 18.5 kg/m^2^; healthy weight BMI = 18.5–24.9 kg/m^2^; overweight BMI = 25–29.9 kg/m^2^; obese BMI = >30 kg/m^2^ [33]

**Table S2.** Associations between FT rank, energy and macronutrient intakes from the 24-hour food recall in excluded male participants.

|  | Nutrient Intake (g) |  | Nutrient Intake (g): Adjusted for Energy |  | % Energy from Nutrients |
| --- | --- | --- | --- | --- | --- |
| *n*=23 | $\hat{\beta}$ (95% CI) |  | $\hat{\beta}$ (95% CI) |  | $\hat{\beta}$ (95% CI) |
| Energy (MJ) | 0.3 (0.1, 0.5) |  | - |  | - |
| Total Fat | 0.016 (0.001, 0.031)* |  | 0.006 (-0.006, 0.018) |  | -0.051 (-0.120, 0.019) |
| Sat. Fat | 0.035 (0.007, 0.064)* |  | 0.027 (0.003, 0.050)* |  | 0.010 (-0.275, 0.295) |
| Mono. Fat | 0.036 (-0.009, 0.081) |  | 0.029 (-0.010, 0.068) |  | -0.102 (-0.261, 0.058) |
| Poly. Fat | 0.030 (-0.117, 0.177) |  | 0.044 (-0.072, 0.159) |  | -0.216 (-0.650, 0.219) |
| Protein | 0.021 (-0.010, 0.052) |  | 0.025 (-0.001, 0.050) |  | 0.010 (-0.193, 0.213) |
| CHO | 0.011 (0.002, 0.020)* |  | 0.012 (0.003, 0.021)* |  | -0.021 (-0.201, 0.159) |
| Alcohol | 0.013 (-0.019, 0.045) |  | -0.006 (-0.032, 0.021) |  | 0.024 (-0.049, 0.097) |

$\hat{\beta}$, estimated slope obtained under a mixed model including twin pair as a random effect; regression analysis was adjusted for age; CI, confidence intervals; Sat. Fat, saturated fat; Mono. Fat, monounsaturated fat; Poly. Fat, polyunsaturated fat; CHO, carbohydrate

**Table S3.** Categories for food items assessed in the Food Frequency Questionnaire based on the AHS 2011-2013 classification system [31]

| **Category** | **Two-digit Major Food Groups** | **Food Frequency Questionnaire items** |
| --- | --- | --- |
| Grain & Cereal | Cereals and cereal products | *“white bread, toast or rolls”, “wholemeal/mixed grain bread, toast or rolls”, “English muffin, bagel and crumpet”, “dry or savoury biscuits, crispbread, crackers”, “muesli”, “cooked porridge”, “breakfast cereal”, “rice (including white or brown)”, “pasta (including filled), noodles”* |
| Meat & Meat Alternatives | Fish and seafood products and dishes | *“canned tuna, salmon, sardines”, “fish – steamed, baked, grilled”, “other seafood (eg, prawns)”* |
|  | Egg products and dishes | *“egg”* |
|  | Meat, poultry and game products and dishes | *“mince dishes (eg rissoles, meat loaf)”, “mixed dishes with beef, veal (eg, casserole, stir-fry)”, “beef, veal – roast, chop or steak”, “mixed dishes with lamb (eg, casserole, stir-fry)”, “lamb – roast, chop or steak”, “mixed dishes with pork (eg, casserole, stir-fry)”, “pork – roast, chop or steak”, “liver (including pate)”, “mixed dishes with chicken, turkey, duck (eg, casserole, stir-fry)”, “chicken, turkey, duck – roast, steamed, BBQ”,* |
|  | Seed and nut products and dishes | *“nuts”, “peanut butter, other nut spreads”* |
|  | Dairy and meat substitutes | *“soybeans or tofu”* |
| Vegetables | Vegetable products and dishes | *“green/mixed salad (including lettuce, tomato, etc.)”, “in a sandwich”, “as a side salad/with a main meal”, “stir-fried or mixed vegetables”, “vegetable casserole”, “potato – boiled, mashed, baked”, “pumpkin”, “sweet potato”, “peas”, “green beans”, “silverbeet, spinach”, “broccoli”, “cauliflower”, “Brussel sprouts, cabbage, coleslaw”, “carrots”, “zucchini, eggplant, squash”, “capsicum”, “sweetcorn, corn on the cob”, “mushrooms”, “tomatoes”, “lettuce”, “celery, cucumber”, “onions or leeks”, “baked beans”, “other beans, lentils”* |
| Fruit | Fruit products and dishes | *“apple or pear”, “orange, mandarin or grapefruit”, “banana”, “peach, nectarine, plum or apricot”, “mango or paw-paw”, “pineapple”, “grapes or berries”, “melon (eg, watermelon, rockmelon, honeydew melon)”* |
| Low-Fat Dairy | Milk products and dishes (dairy milk^1^) | *“milk as a drink”, “milk on breakfast cereals”, “milk in hot beverages (eg, in coffee, tea)”* |
|  | Milk products and dishes (yoghurt^1^) | *“yoghurt, plain or flavoured (including fromage frais)”* |
| High-Fat Dairy | Milk products and dishes (cream^1^) | *“cream or sour cream”* |
|  | Milk products and dishes (cheese^1^) | *“cheddar and other cheeses”, “cottage or ricotta cheese”* |
|  | Milk products and dishes (frozen milk products^1^) | *“ice-cream”* |
|  | Milk products and dishes (flavoured milks and milkshakes^1^) | *“flavoured milk drink (eg, milkshake, iced coffee, hot chocolate)”* |
| Discretionary Food | Meat, poultry and game products and dishes^2^ | *“sausage, frankfurter”, “bacon”, “ham”, “luncheon meats, salami”, “hamburger”* |
|  | Fish and seafood products and dishes^2^ | *“fish – fried”* |
|  | Vegetable products and dishes^2^ | *“hot chips”* |
|  | Cereal based products and dishes | *“cakes, sweet muffins, scones or pikelets”, “sweet pies or sweet pastries”, “other puddings or desserts”, “plain sweet biscuits”, “cream or chocolate biscuits” , “meat pie, sausage rolls or other savoury pastries”, “pizza”,* |
|  | Savoury sauces and condiments | *“oil and vinegar dressing”, “mayonnaise or other creamy dressing”,* |
|  | Snack foods | *“potato chips, corn chips, Twisties, etc.”* |
|  | Sugar products and dishes | *“jam, marmalade, syrup or honey”* |
|  | Confectionary and cereal/nut/fruit/seed bars | *“chocolate (including chocolate bars, eg Mars bars)”, “other confectionary”* |
|  | Miscellaneous | *“Vegemite, Marmite, Promite”* |
| Alcoholic Beverages | Alcoholic beverages | *“beer – low alcohol”, “beer – ordinary”, “red wine”, “white wine or champagne/sparkling wine”, “wine cooler”, “sherry or port”, “spirits, liqueurs”* |

^1^Three-digit sub-major food group
^2^Products flagged as discretionary based on the Australian Health Survey – Discretionary Food List [31]

**Table S4.** Associations between FT rank, energy and macronutrient intakes in women excluding low-energy reporters.

|  | Nutrient Intake (g) |  | Nutrient Intake (g): Adjusted for Energy |  | % Energy from Nutrients |
| --- | --- | --- | --- | --- | --- |
| *n*=53 | $\hat{\beta}$ (95% CI) |  | $\hat{\beta}$ (95% CI) |  | $\hat{\beta}$ (95% CI) |
| Energy (MJ) | 0.1 (-0.2, 0.4) |  | - |  | - |
| Total Fat | 0.017 (-0.013, 0.046) |  | 0.017 (-0.012, 0.046) |  | 0.087 (-0.046, 0.220) |
| Sat. Fat | 0.061 (-0.014, 0.136) |  | 0.063 (-0.009, 0.136) |  | 0.293 (-0.021, 0.606) |
| Mono. Fat | 0.041 (-0.022, 0.103) |  | 0.042 (-0.021, 0.104) |  | 0.143 (-0.137, 0.423) |
| Poly. Fat | -0.021 (-0.158, 0.117) |  | -0.022 (-0.146, 0.102) |  | -0.022 (-0.490, 0.445) |
| Protein | 0.009 (-0.013, 0.032) |  | 0.009 (-0.013, 0.031) |  | 0.014 (-0.161, 0.188) |
| CHO | 0.000 (-0.010, 0.010) |  | -0.001 (-0.011, 0.009) |  | -0.059 (-0.166, 0.049) |
| Alcohol | 0.009 (-0.024, 0.042) |  | 0.007 (-0.030, 0.044) |  | 0.031 (-0.059, 0.120) |

$\hat{\beta}$, estimated slope obtained under a mixed model including twin pair as a random effect; regression analysis was adjusted for age; CI, confidence intervals; Sat. Fat, saturated fat; Mono. Fat, monounsaturated fat; Poly. Fat, polyunsaturated fat; CHO, carbohydrate
